# Beta-Secretase 1 Underlies Reactive Astrocytes and Endothelial Disruption in Neurodegeneration

**DOI:** 10.3389/fncel.2021.656832

**Published:** 2021-05-06

**Authors:** María Victoria Chacón-Quintero, Lina Gisela Pineda-López, Carlos Andrés Villegas-Lanau, Rafael Posada-Duque, Gloria Patricia Cardona-Gómez

**Affiliations:** ^1^Neuroscience Group of Antioquia, Faculty of Medicine, University of Antioquia, Cellular and Molecular Neurobiology Area, Medellin, Colombia; ^2^Institute of Biology, Faculty of Exact and Natural Sciences, University of Antioquia, Medellin, Colombia; ^3^Neurobank, Faculty of Medicine, SIU, University of Antioquia, Medellin, Colombia

**Keywords:** BACE1, reactive astrocytes, endothelial disruption, neurodegeneration, neurovascular unit

## Abstract

Dysfunction in the neurovascular unit (NVU) is a key component in the progressive deterioration of Alzheimer’s disease (AD) and is critical in vascular dementia. Recent studies have shown that inflammation plays early and perhaps causal roles in the pathogenesis of AD related to NVU damage, possibly in part by overactivating the aspartic acid protease activity of β-site amyloid precursor protein-cleaving enzyme 1 (BACE1), which until now has almost solely been studied in the context of the β-amyloid cascade. In this study, we analyzed the relationship of BACE1 with astrocytes and blood vessels in human brains with sporadic and familial dementia [Autosomal dominant cerebral arteriopathy with subcortical infarcts and leukoencephalopathy (CADASIL), sporadic Alzheimer’s disease (SAD), and familial Alzheimer’s disease (FAD)] and how BACE1 inhibition affects astrocytes and endothelial cells under conditions of glutamate toxicity. Our results show increased BACE1, PHF (Paired helical filaments)-tau and GFAP (Glial Fibrillary Acid Protein) immunoreactivity (IR) in the CA1 hippocampal regions of FAD and SAD brains. Furthermore, BACE1 immunoprecipitated with GFAP in tissue samples from all study cases, but their immunofluorescence close to (10 μm^3^) or overlapping blood vessels was only increased in FAD and SAD brains, and PHF-tau was present around the vessels mainly in FAD brains. Interestingly, the increased BACE1 levels were associated with reactive astrocytes, characterized by morphological changes and upregulation of GFAP under pathological and stressful conditions, and endothelial disruption by glutamate excitotoxicity, and these effects were reversed by BACE1 inhibition; further, BACE1-inhibited astrocytes protected endothelial cell integrity by preserving zonula occludens-1 (ZO-1) distribution and decreasing the expression of inflammatory markers. Taken together, these findings suggest that BACE1 dysregulation in astrocytes may have a role in the alterations in NVU integrity implicated in neurodegeneration.

## Introduction

The blood brain-barrier (BBB) is a specialized structure which allow the exchange of molecules between peripheral circulatory system and the central nervous system (CNS) (Zenaro et al., 2016). The properties of the BBB are regulated by the function of the neurovascular unit (NVU), which is composed of endothelial cells (ECs) in contact with the basal lamina, pericytes, astrocytes, and neurons and is closely associated with the extracellular matrix (Posada-Duque et al., 2014; Villabona-Rueda et al., 2019). Its importance and definition emerged from “First stroke progress review group meeting of the National Institute of Neurological disorders and stroke of the NIH” in July 2001, as the coupling of the cerebral blood flow and the neural activity (Iadecola, 2017), allowing the bi-directional communication between neurons and microvessels, which was comprehensively analyzed in stroke research (del Zoppo, 2010). Astrocytes are closely related to the ECs that comprise the BBB and support its functions through the release of trophic factors and maintain the integrity of tight junctions such as Claudin-5 (CLDN5) and ZO-1 (Barrier et al., 1997; Gaillard et al., 2000; Sofroniew and Vinters, 2010; Alvarez et al., 2011). These astroglial cells maintain the homeostasis of the brain parenchyma by regulating energy and metabolism, blood flow and synapse function. NVU components are susceptible to damage from high glutamate concentrations; specifically, in ECs, glutamate triggers apoptosis mediated by oxidative stress (Parfenova et al., 2006; Jean et al., 2013) and, in astrocytes, astrogliosis (Pekny et al., 2018). Reactive astrocytes propagate pro-inflammatory signals in addition to activating catabolic processes and triggering apoptosis; however, they also facilitate the uptake and synthesis of neurotransmitters, induce angiogenesis and mediate antioxidation, thus contributing to neuroprotection. The roles of reactive astrocytes depend on the profile of genes that are differentially expressed and the postinjury time (Becerra-Calixto and Cardona-Gómez, 2017).

BACE1 is expressed in the ECs of the human BBB; it has also been shown to be predominantly localized to the membrane with an abluminal distribution of BACE1 in brain microvessels having a role in the vascular aspects of Alzheimer’s disease, particularly in the development of amyloid cerebral angiopathy (Devraj et al., 2016). BACE1 is a protease found in lipid rafts localized in endosomes and to a lesser extent in the trans-Golgi network (Koelsch, 2017). It has been characterized by its participation in amyloidosis and vascular deterioration. Although its expression was initially described in neurons, it has since been reported to be expressed in astrocytes and ECs at higher levels during stressful events such as ischemic stroke, in which a pro-inflammatory microenvironment is generated (Hartlage-Rübsamen et al., 2003; Bettegazzi et al., 2011; Zhao et al., 2011; Bulbarelli et al., 2012; Devraj et al., 2016).

Cerebrovascular dysfunction has been documented in vascular-type dementia, a group of heterogeneous brain disorders in which cognitive decline is associated with pre-existing pathological conditions in the cerebral vasculature (Costantino Iadecola, 2013). Some recent studies suggest that NVU dysfunction is not only a key component in the progressive deterioration of Alzheimer’s pathology and critical in vascular dementia but also an early mediating factor in the initiation of the neurodegenerative cascades observed in both diseases (Zenaro et al., 2016; Nation et al., 2019). Consequently, an understanding of the cellular mechanisms involved in NVU dysfunction would be an extraordinary tool to improve our knowledge of the pathobiology of dementias, which would lead to the development of new therapeutic approaches (ElAli, 2014). Therefore, it is necessary to advance the understanding of BACE1 dysregulation and its role in the alterations of components of the NVU in neurodegenerative processes. To pursue this aim, we analyzed the relationship of BACE1 with astrocytes and blood vessels in human brains from individuals with sporadic and familial dementia (CADASIL, SAD, and FAD) and how BACE1-inhibited astrocytes acting on endothelial cells in a glutamate toxic environment. We found an increased expression of BACE1 in reactive astrocytes associated with hyperphosphorylated tau and located close to or overlapping with blood vessels in the AD cases. In addition, BACE1-inhibited astrocytes reduced the induced damage by glutamate in ECs in an *in vitro* model. Therefore, we propose that an overload of BACE1 in reactive astrocytes close to vessels is a triggering factor for neurodegeneration in AD.

## Materials and Methods

### Human Brain Tissue

Postmortem hippocampal brain tissue from 5 cases of FAD (PSEN1 E280A mutation), 5 cases of SAD, 5 cases of CADASIL and 5 healthy age-matched individuals (Control) obtained from the Neurobank of the University of Antioquia were included in this study. Informed consent was obtained for the research, and the study was approved by the bioethics committee for human studies at the University of Antioquia. The brain tissue was fixed in tissue blocks by immersion in a 4% paraformaldehyde solution in 0.1 M phosphate buffer (PB) (pH 7.4) at 4°C for 7 days. After 7 days, the tissues were subjected to a sucrose gradient of 7, 25, and 30% in PB. The hippocampal tissue was embedded in isopentane and subsequently stored at −80°C. Then, 50-μm sections were cut in a cryostat (Leica CM1850). The cases used to carry out this study are shown in Table 1.

**TABLE 1:** Study cases used according to pathological condition.

Nbiol code	Neuro Biobank code	Condition	Gender	Onset age	Age of death	CERAD	Braak	Thal
C2	240	Control -Healthy	F	Does not apply	67	0	1	0
C3	243	Control -Healthy	F	Does not apply	75	0	0	0
C4	262	Control -Healthy	M	Does not apply	69	A	1	2
C5	319	Control -Healthy	M	Does not apply	61	0	0	0
C7	342	Control -Healthy	F	Does not apply	44	0	0	0
F2	127	FAD (E280A)	F	49	62	B	4	5
F3	322	FAD (E280A)	F	44	50	C	6	5
F5	328	FAD (E280A)	F	50	63	C	6	5
F6	335	FAD (E280A)	M	49	59	C	6	5
F7	339	FAD (E280A)	F	51	65	B	5	5
SI	99	Late SAD	F	82	92	C	5	4
S2	112	Late SAD	F	62	74	B	5	4
S3	117	Early SAD	F	55	76	B	4	5
S6	332	SAD	F	81	94	B	4	5
S10	338	SAD	F	92	98	A	3	3
Al	118	CADASIL	F	52	65	B	1	2
A2	147	CADASIL	F	35	45	0	0	0
A3	160	CADASIL	F	32	49	0	0	0
A4	201	CADASIL	M	41	59	0	0	0
A5	321	CADASIL	F	55	78	0	0	0

*We used postmortem brain samples from *n* = 5 CNT, *n* = 5 SAD, *n* = 5 FAD (PSEN1 E280A mutation), and *n* = 5 CADASIL (Notch 3 mutation) cases obtained from the University of Antioquia Neurobank. The cases were classified as healthy controls (C), familial Alzheimer’s (F), sporadic Alzheimer’s (S), or CADASIL (A). The table includes the age of onset, age of death, postmortem index as the time between the patient’s death and sampling, Consortium to Establish a Registry for Alzheimer’s Disease (CERAD) Braak stage to classify the degree of AD, and Thal phase to show the intersection of tau and amyloid β. NA, not available; NAP, not applicable; B, histological findings suggest the diagnosis of AD; C, histological findings indicate the diagnosis of AD.*

### Immunohistochemistry

Floating human hippocampal tissue sections (50 μm thick) were exposed to epitopes in 98% formic acid (Sigma, 100264) for 5–6 min at 85°C, and 30% Triton ^TM^ X-100 (Sigma, 93443) in PB for 5 min. Endogenous peroxidase activity was blocked using 1:1 methanol with 1% hydrogen peroxide in PB for 20 min at room temperature. Non-specific antibody binding sites were blocked with 1% bovine serum albumin (BSA) (Sigma-Aldrich) and 0.3% Triton X-100 in PB for 1 h. The sections were incubated with rabbit anti-BACE1 (ab108394, Abcam, 1:100), mouse-anti-human phospho-tau recognizing phosphorylated serine 202 and threonine 205 from tau. (PHF-tau, MN1020, Thermo Fisher Scientific, 1:1000), mouse anti-glial fibrillary acid protein (GFAP, G3893, Sigma-Aldrich, 1:250), or rabbit anti-vimentin (ab137321, Abcam, 1:250) primary antibody in PB with 0.3% Triton X-100 and 0.3% BSA at 4°C for 3 nights. The slices were then incubated with anti-mouse (31800, Invitrogen, 1:250) or anti-rabbit (B2770, Invitrogen, 1:250) biotinylated secondary antibody for 1 h and then incubated with the avidin biotin complex (ABC Standard Peroxidase Staining Kit, Pierce #32020, 1:250 reagent A:B) for 1 h. Staining was performed using diaminobenzidine (DAB, 12623957, Thermo Fisher Scientific) in 1% hydrogen peroxide. The slices were mounted and dried on slides, dehydrated by an alcohol gradient, and covered with the Consul-Mount mounting solution (Thermo Fisher Scientific; 9990440).

### Microscopy, Image Processing, and Analysis

The tissue IR was analyzed at 10× (air objective, NA 0,25, Nikon) magnification by light microscopy (Nikon Epsilon E200) with a Nikon digital sight DS-L1 camera. For each slide, two consecutive images were taken allowing encompass all the extension of CA1 area for its analysis. The images were transformed to 8-bit and then analyzed using the binary threshold in the ImageJ software (NIH ImageJ). To calculate the total stained area, segmentation of all images was performed using intensity thresholding.

### Immunofluorescence

For immunofluorescence staining, a procedure similar to that described above for immunohistochemistry was performed. After antigen retrieval with formic acid, tissue and background autofluorescence were blocked with 0.1% Sudan Black B (Sigma, S0395) in 70% ethanol for 10 min. Preincubation was performed with 1% BSA and 0.3% Triton, and the sections were then incubated with primary antibody mixtures for triple immunofluorescent staining. The first antibody mixture contained rabbit anti-GFAP (PA516291, Invitrogen, 1:250), mouse-anti-phospho-PHF-tau (MN1020, Thermo Fisher Scientific, 1:250) and DyLight 649-labeled *Ulex Europaeus agglutinin* (UAE) lectin (Vector Labs; DL-1068; 1:750), and the second antibody mixture contained anti-BACE1 (ab108394, Abcam, 1:100), mouse anti-GFAP (G3893, Sigma-Aldrich, 1:250) and DyLight 649-labeled UAE lectin. Both antibody mixtures were prepared in 0.3% Triton X-100 and 0.3% BSA in PB, and the sections were incubated in each mixture at 4°C for 3 nights. The fluorescent anti-rabbit antibodies Alexa 488 (A11008, Invitrogen, 1:750), anti-mouse Alexa 594 (A11005, Invitrogen, 1:750), anti-rabbit Alexa 594 (A11012, Invitrogen, 1:750) and anti-mouse Alexa 488 (A11001, Invitrogen, 1:750) were incubated for 1 h. The tissue sections were washed three times for 5 min and then mounted on slide plates and sealed on coverslips with FluorSave (Millipore; 345789). Sections incubated in parallel to the sections described above but without the primary antibodies were included as negative controls for the background binding of the secondary antibody and to discriminate autofluorescence. The omission of the primary antibodies did not produce staining.

### Confocal Microscopy

The triple-stained mounted tissue sections were analyzed by a confocal laser scanning microscope (FV1000 Olympus, Japan) using a 60X objective (immersion oil, NA 1.42) and the Olympus FluoView program. A total of two random fields in the CA1 area were imaged for each section. For each experimental case, 21 consecutive individual images were obtained at 0.5 μm intervals in all channels along the *Z* axis of the sample. The image acquisition parameters remained the same between the samples. 16-bit TIFF images of 1024 x 1024 pixels (105.47 x 105.47 μm) were obtained with an XY pixel size of 103 nm and 500 nm between Z-sections. The confocal images were deconvolved, processed, and segmented for quantifying the Z stack signal represented as volumetric information; also, maximum projection images were generated for each field for illustrative purposes. The images were deconvolved using the Huygens Professional 19.10 software (Scientific Volume Imaging B.V.). After, these were transformed to 8-bit and then processed and analyzed by the FIJI software (Image J NIH). Then, immunofluorescent signals were segmented using machine learning and intensity thresholding by Otsu algorithm, to standardize the signals in all the images. The total area of staining and the areas of colocalization were measured. Colocalizing signals were identified using the algorithm and from the image calculator tool. A distance map tool from the 3D suite plugin (Ollion et al., 2013) was used to isolate signals located between 0 and 10 μm from the vessel surface to characterize the association of astrocytic endfeet with the vessels and the association with PHF-tau and BACE1 in the CA1 area. Z projections of the deconvolved images were made using the max intensity option, while segmented images were projected using the standard deviation.

### Immunoprecipitation

To confirm the relationship between both GFAP^+^ astrocytes and BACE1, we immunoprecipitated BACE1 and then the co-immunoprecipitated GFAP. Briefly, samples were lysed in 10 mM Tris (pH 7.4), 100 mM NaCl, 1 mM EDTA, 1 mM EGTA, 10% glycerol, 1% NP40, 1 nM orthovanadate, 5 mM NaF, 1 mM phenylmethylsulfonyl fluoride with a protease inhibitor cocktail (Sigma-Aldrich). The lysates were clarified by centrifugation at 14,000 rpm for 5 min. A Pierce Protein Quantification Assay was performed, and then 200 μg total protein was incubated overnight at 4°C in the presence of the anti-BACE1 antibody (ab108394, Abcam, 1:100). Protein G Sepharose beads were added, and the samples were incubated for an additional 2 h at room temperature. The immune complexes were washed three times using immunoprecipitation lysis buffer before SDS-PAGE and immunoblotting. The proteins were separated using 10% SDS-PAGE, transferred to nitrocellulose membranes (Amersham) and probed with mouse anti-GFAP antibody (G3893, Sigma-Aldrich, 1:250). Whole lysates were used as positive controls, and incubation with an IgG peptide (395040065, Thermo, 1:500) was used as a negative control for immunoprecipitation. The blots were visualized using the Odyssey Infrared Imaging System (LI-COR Biosciences). To minimize interassay variation, samples from all experimental groups were run in parallel.

### bEnd.3 Cell Line Cultures

The bEnd.3 (ATCC CRL-2299) murine cell line was used as an endothelial cell model as we described previously (Becerra-Calixto et al., 2018). The bEnd.3 cells were thawed in DMEM (Sigma-Aldrich) supplemented with 20% fetal bovine serum (FBS, Eurobio) and 1% penicillin–streptomycin (Gibco). After 24 h, the medium was replaced with the maintenance medium [DMEM supplemented with 10% fetal bovine serum and 1% penicillin–streptomycin (Gibco)]. The cells were incubated at 37°C in 5% CO2. To perform the subcultures, the cells were trypsinized using a 0.25% trypsin/EDTA mixture (Gibco) for 5 min and subcultured in 12-well plates at a density of 2.5 × 10^5^ cells per well.

### Astrocyte Primary Cultures

Astrocytes were obtained from primary cultures of cortical astrocytes from Wistar rat brains extracted on postnatal day 1 or 2 as we described previously (Posada-Duque et al., 2015). The cortex was enzymatically dissociated with 0.25% trypsin/EDTA mixture (Gibco, 15400054) during 15 min, cultured in T75 flasks (surface area 75 cm^2^) and maintained in DMEM (Sigma-Aldrich) supplemented with 10% FBS and 1% penicillin–streptomycin (Gibco). The cells were incubated at 37°C in 5% CO_2_. The culture medium was changed every 2 days. From day *in vitro* (DIV) 8 to DIV 10, the flasks were shaken at 350 rpm for a sequence of 6, 18, and 24 h to minimize the amount of microglia and oligodendrocytes. Then, the cells were trypsinized using a 0.25% trypsin/EDTA mixture (Gibco) for 5 min and subcultured in 12-well plates at a density of 7.5 × 10^4^ cells per well.

### bEnd.3 and Astrocyte Coculture

Astrocytes were subcultured on DIV 10 on coverslips in 12-well plates. The bEnd.3 cells were thawed and subcultured on gelatinized coverslips with four paraffin dots at the ends until DIV 15 when the coculture was performed. The co-culture assembly consisted in superimposing one coverslip on the other so that both cell types are close and share the culture medium, the paraffin dots allow that there is no direct contact between the cells and therefore, without mechanical damage between them. On DIV 21, the coculture was disassembled to inhibit BACE1 only in astrocytes, and 24 h later, the coculture was reassembled and treated with glutamate (Becerra-Calixto et al., 2018). On DIV 23, the culture medium was collected to measure cytotoxicity, and both cell types were fixed for immunofluorescence staining.

### Glutamate-Induced Toxicity Assay and Inhibitor Treatment

#### bEnd.3

The bEnd.3 cell cultures were pretreated on DIV 8 with β-secretase inhibitor IV (CAS 797035-11-1, Merck) at 1 μM. Twenty-four hours later, the cells were treated with glutamate at 125 μM for 20 min and were subsequently treated with the inhibitor again. On DIV 10, the culture medium was collected to measure the cytotoxicity, and the cells were fixed for immunofluorescence staining.

#### Primary Astrocytes

The astrocytes were pretreated on DIV 21 with β-secretase inhibitor IV at 1 μM. Twenty-four hours later, the cells were treated with glutamate at 125 μM for 24 h and were subsequently treated with the inhibitor again. On DIV 23, the culture medium was collected to measure the cytotoxicity, and the cells were fixed for immunofluorescence staining.

#### Coculture

In the coculture, BACE1 was inhibited in the astrocytes at the previously described concentration; however, both cell types were exposed to glutamate treatment for 20 min. On DIV 23, the medium was collected to measure the cytotoxicity, and the coculture was disassembled to fix both cell types.

### LDH Cytotoxicity Assay

The cytotoxicity was measured by evaluating the percentage of LDH release with the Roche LDH Cytotoxicity Detection Kit. The assay was performed by mixing the media with a mixture of the two kit solutions, incubating the combined mixture for 30 min in the dark, and measuring the absorbances of the samples at 490 nm in a microplate reader. The percentage of LDH release was calculated using the following formula: %*LDHrelease*((A−*lowcontrol*)/(*highcontrol*−*lowcontrol*)) 100, where A was the absorbance indicating the LDH activity level, the low control was the LDH activity of basal release from untreated cells and the high control was the measure of maximum LDH release from the cells, which was obtained from cells treated with 1% Triton X-100.

### *In vitro* Immunofluorescence

The cell cultures were fixed with 4% paraformaldehyde in cytoskeleton buffer with sucrose (CBS) (Posada-Duque et al., 2017). Autofluorescence was eliminated using 50 mM ammonium chloride (NH4Cl) for 10 min. The cells were permeabilized with 0.2% Triton. X-100 prepared in CBS and then treated with a blocking solution (2.5% FBS in CBS). The cultures were incubated overnight at 4°C with mouse primary antibodies against mouse CLDN5 (Invitrogen, 1:750), mouse GFAP (Sigma-Aldrich, 1:750), rabbit BACE1 (Abcam, 1:500) and mouse IL-1β (Abcam, 1:500). Subsequently, the cultures were incubated for 1 hour with Alexa 594- or Alexa 488-tagged secondary antibodies (Molecular Probes, 1:500), and the nuclei and cytoskeleton were stained with Hoechst 33258 (Invitrogen, 1:5000) and phalloidin conjugated with Alexa 594 or Alexa 488 (1:500, Molecular Probes). Then, serial washes with PBS (phosphate-buffered saline) were performed, and the coverslips with the immunolabeled cells were fixed to slides with FluorSave. The cells were observed under an Olympus IX 81 epifluorescence microscope, and the images were captured with an oil immersion objective (60X, NA 1.42) and then processed.

### Morphological Analysis

#### Condensed Nuclei

The average diameter of each nucleus was quantified by automatic measurements using the “Count and measure objects” tool of the Image-Pro Plus software. Nuclei with diameters between 3.0 and 6.0 μm were defined as condensed. The percentage of condensed nuclei was calculated using the following formula: Percentage of condensed nuclei= [(condensed nuclei/(condensed nuclei + normal nuclei)]×100.

#### Number and Area of Gaps

The gaps are visualized as spaces between ECs indicate damage in the cell monolayer. This term was defined as: disruption of endothelial cells involving extravasation (Hirata et al., 1995) and inflammation more than cell death (Israelov et al., 2020). To determine the number of these spaces, F-actin signals were segmented using machine learning and intensity thresholding by Otsu algorithm, to standardize the signals in all the images, a binary image of each analyzed field was generated using the “Magic Wand” tool of the Adobe Photoshop software (González-Molina et al., 2021). With the images obtained, the number and area of gaps were measured using the “Count and measure objects” tool of the Image-Pro Plus software.

#### Fluorescence Intensity Quantification

Fluorescence intensity (FI) measurements for each protein were obtained from the color channels by calculating the intensity using the “Measure” tool, which was relativized with the cell area. These analyses were performed using ImageJ (NIH software).

#### Fluorescence Profiles

To determine the CLDN5 distribution, a fluorescence profile was established by drawing a white line of 50 μm through the cells (tracing it through the membrane, the cytoplasm and the nucleus) using the “Line profile” tool of the Image-Pro Plus software. The fluorescence profiles capturing the changes per channel of 20 cells were made for each treatment and the most representative ones were chosen for the figures.

### Statistical Analysis

The statistical analysis of the human data was performed with the GraphPad Prism software (version 6.0). Data were plotted as the mean ± standard error of the mean (SEM) for the quantitative variables. A variance homogeneity test was performed, and multivariate analyses were performed for one-way ANOVA parametric data, post hoc Kruskal-Wallis tests and Student’s t tests. Values of *p* < 0.05 were considered significant.

For the *in vitro* analyses, the parametric data were compared using one-way ANOVA followed by the Tukey-Kramer post hoc test to identify the means that were significantly different from each other. The nonparametric data were compared using the Kruskal-Wallis test and Dunn’s test with Bonferroni correction. The results were considered significant when *p* < 0.05 (^∗^ indicates *p* < 0.05, ^∗∗^ indicates *p* < 0.01, and ^∗∗∗^ indicates *p* < 0.001). The data analysis was performed using the R version 3.4.4 software (R Core Team, 2018).

## Results

### BACE1 Expression Is Increased in the PHF-tau^+^ CA1 Area and Subiculum of Sporadic and Familial Alzheimer’s Disease Brains

**FIGURE 1:**
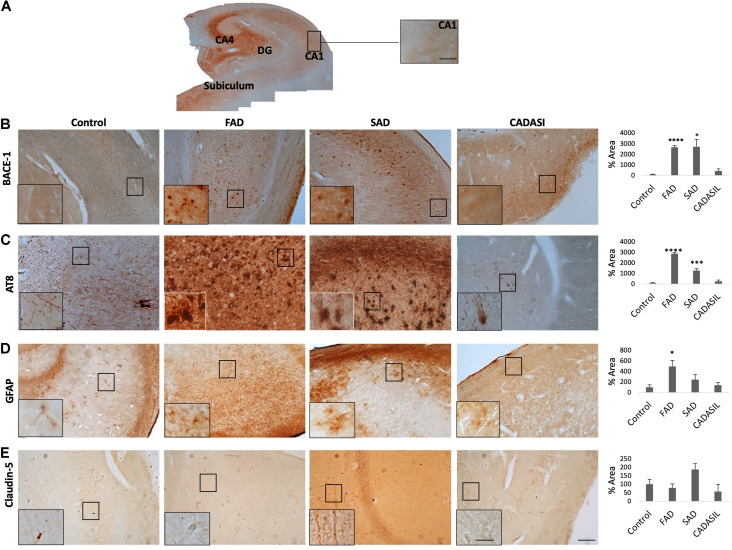
BACE1, PHF-tau, GFAP, and CLDN5 immunoreactivities in the CA1 area of dementia brains. **(A)** Representative image of location in human Control hippocampus of area ca1. Magnification: 10×. Scale bar: 25 μm. **(B)** Representative images of the BACE1, **(C)** PHF-tau, **(D)** GFAP, and **(E)** CLDN5 immunoreactivities in the CA1 area of human hippocampal tissue. Magnification: 10×. Scale bar: 50 μm. Inset: 25 μm scale bar. The values in the bar graph are expressed as a densitometric percentage of the BACE1 IR in the CA1 area. FAD, familial-type Alzheimer’s disease (presenilin 1 mutation E280A); SAD, sporadic Alzheimer’s disease; CADASIL, autosomal dominant cerebral arteriopathy with subcortical infarcts and leukoencephalopathy. The data are expressed as the means ± SEM. *n* = 4. ^∗^*p* < 0.05; *** *p* < 0.001; ****p < 0.0001.

We analyzed the IR of BACE1, PHF-tau, GFAP and CLDN5 in human hippocampal tissue samples from patients with FAD, SAD, and CADASIL and from healthy control samples (Figures 1A–D). In the CA1 area, we found a significant increase in BACE1 IR in the FAD and SAD groups compared to that in the control group, while no significant differences were found in the CADASIL group (Figure 1A). The PHF-tau IR was increased in the CA1 area in the FAD and SAD groups and was significantly greater in the CA1 area in the FAD group than in the control group. The PHF-tau IR in the CADASIL group did not differ from that in the control group (Figure 1B). On the other hand, the GFAP IR was significantly higher in the FAD group than in the control group, and nonsignificant differences were observed in the SAD and CADASIL groups (Figure 1C). Finally, the CLDN5 data suggest a clear tendency toward increased expression in the CA1 area in the SAD group compared to that in the control group (Figure 1D), whereas the other groups did not show any differences.

Furthermore, we analyzed the IR of subiculum and CA4 areas in the same slices of human hippocampal tissues from FAD, SAD, CADASIL, and healthy control cases (Supplementary Figures 1A, 2A, respectively). In subiculum we detected a significant increase of BACE1 in all dementia cases being significant in FAD and CADASIL respect to the control group (Supplementary Figure 1B). Additionally, the PHF-tau IR presented an augment in FAD and SAD compared respect to the control, without change in CADASIL (Supplementary Figure 1C). Moreover, the levels of GFAP IR has a clear tendency to increase in all dementias cases, it was significant higher in FAD (Supplementary Figure 1D). On this way, CLDN5 data suggest a slight augment in the SAD group respect to the control and the other groups (Supplementary Figure 1E).

Inversely, in the CA4 area we found a significant decrease in BACE1 in SAD and CADASIL compared to the control group (Supplementary Figure 2B). Furthermore, PHF-tau IR was increased in FAD and SAD cases, and the increase was significant higher in FAD respect to SAD (Supplementary Figure 2C). However, GFAP and CLDN5 IRs did not present significant changes in CA4 area between the analyzed groups (Supplementary Figures 2D,E).

Finally, we selected the CA1 area for the subsequent analyses as a representative zone of neurodegeneration in the hippocampus.

### BACE1 Expression Is Increased in Reactive Astrocytes Around Blood Vessels, Which Are Associated With Hyperphosphorylated Tau in the Human AD Hippocampus

**FIGURE 2:**
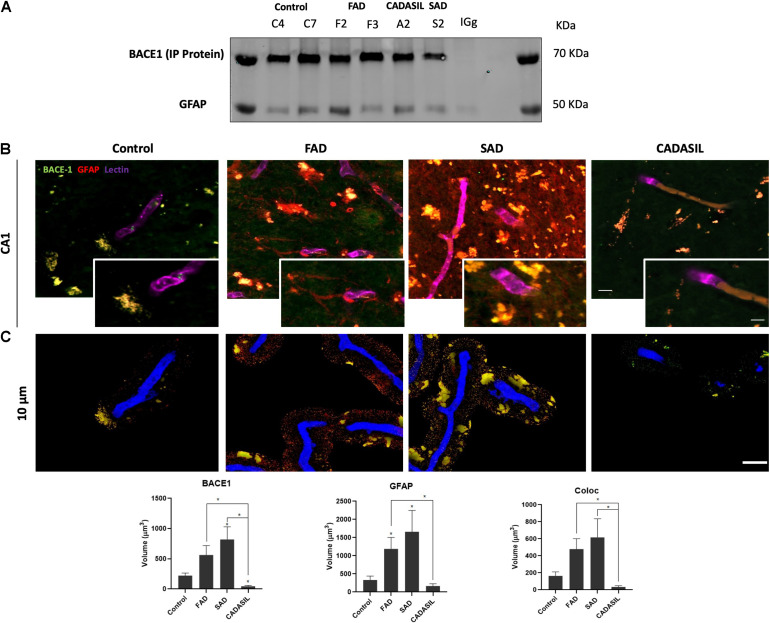
BACE1 association with astrocytes and vessels in dementia brains. **(A)** Representative protein bands from the immunoprecipitation of BACE1 and co-inmunoprecipitation of GFAP are shown. IgG was used as a negative control for immunoprecipitation. **(B)** Z projection of Immunofluorescence of blood vessels triple-stained with DyLight 649- tagged UEA (color-coded in blue), anti-BACE1 antibody probed with Alexa Fluor 488 (green) and reactive astrocytes marked with Alexa Fluor 594 (red) in the hippocampal CA1 area. Magnification: 60x. Scale bar: 25 μm. Insets: 5 μm scale bar in B. **(C)** Z projection of the deconvolved images from the 3D reconstruction of the confocal images at 0 to 10 μm away from the vessel surface. Scale bar: 25 μm. The values represent the volumes in μm^3^ for the levels of BACE1 and GFAP and the colocalization of BACE1 and GFAP. The data are expressed as the means ± SEM. *n* = 4. *p < 0.05.

**FIGURE 3:**
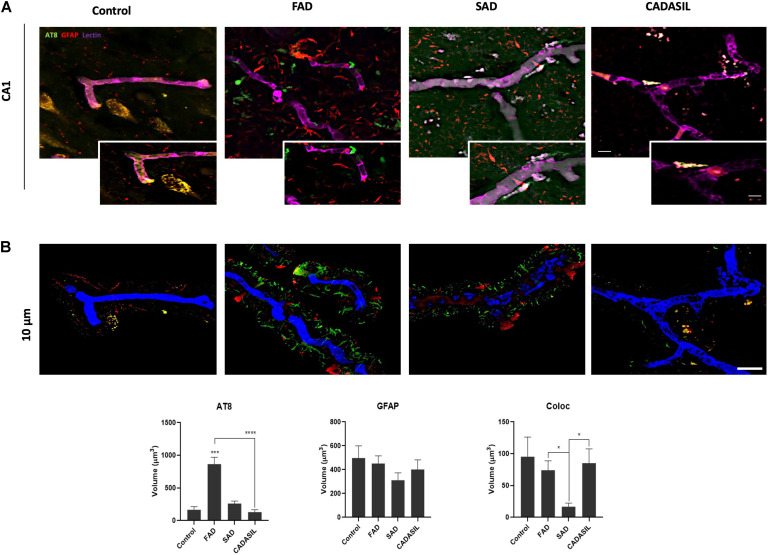
PHF-tau association with astrocytes and vessels in dementia brains. **(A)** Z projection of Immunofluorescence of blood vessels triple-stained with DyLight 649 UEA (blue), PHF-tau antibody probed with Alexa Fluor 488 (green), and reactive astrocytes marked with Alexa Fluor 594 (red) in the hippocampal CA1 area. Magnification: 60x. Scale bar: 25 μm. Insets: 5 μm scale bar in A. **(B)** Z projection of the deconvolved images from the 3D reconstruction of the confocal images in A showing the triple staining present at 0 to 10 μm away from the vessel surface. Scale bar: 25 μm. The quantification of the volume in μm^3^ for the levels of PHF-tau and GFAP and the colocalization between PHF-tau and GFAP. The data are expressed as the means ± SEM. *n* = 4. *p < 0.05; ***p < 0.001; ****p < 0.0001.

After the confirmation of BACE1 and GFAP expressions in the hippocampus, we assessed if both proteins presented association by co-immunoprecipitation, how it had been reported previously (Sil et al., 2020) and if presented differential association between dementias. However, we found the complex in all cases, which at least confirm the presence of BACE1 in GFAP+ astrocytes (Liang et al., 2020; Figure 2A). This finding was confirmed qualitatively by immunofluorescence in the SAD and FAD cases, which showed astrogliosis (increase of reactive astrocytes) (Figure 2B). After, we performed a deeper analysis of the association of BACE1 with GFAP around vessels in the CA1 area of the hippocampus in human brains with dementia. We confirmed a significant increase in the BACE1 and GFAP levels in the FAD and SAD groups compared with those in the control group and found that BACE1 and GFAP colocalized close to (10 μm^3^) or overlapped with vessels mainly in the FAD and SAD groups, although the vessels were disrupted in the SAD group (Figure 2C). In addition, we found a significant increase in PHF-tau close to (10 μm^3^) or overlapping with the vessels, and more of the PHF-tau^+^ cells associated with GFAP+ cells in the FAD group than in the SAD group (Figure 3); both the FAD and SAD groups exhibited a higher association of PHF-tau+ cells with GFAP+ cells than the control and CADASIL groups. Additionally, vessel disruption mainly appeared in the SAD group samples.

### BACE1 Inhibition Protects Endothelial Cell Integrity Under Glutamate Toxicity by Reversing Structural and Inflammatory Damage

**FIGURE 4:**
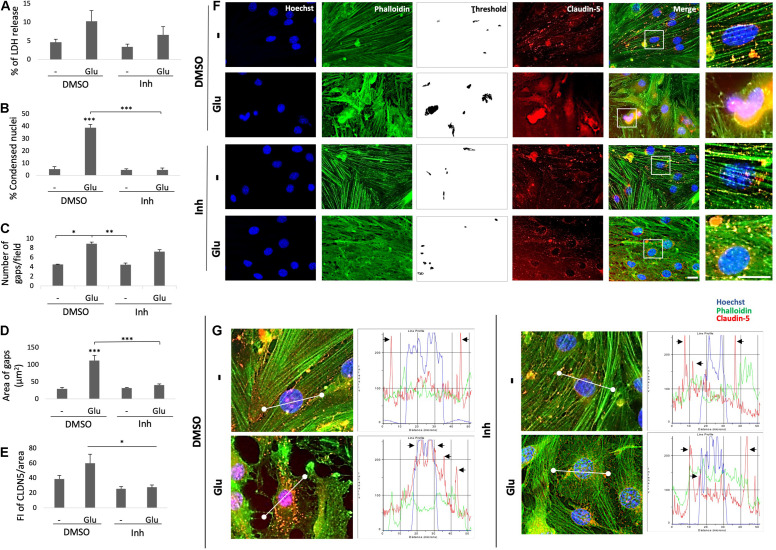
BACE1 inhibition protects ECs from the damaging effects of glutamate. **(A)** Percentage of LDH release by ECs (bEnd.3) after 24 h of treatment. The data are presented as the mean ± SEM of *n* = 7. Kruskal-Wallis test. **(B)** Percentage of condensed nuclei for each treatment. These were quantified from 20 fields per treatment for each n (*n* = 4). The data are presented as the means ± SEM. **(C)** Number of gaps per field from threshold image (gaps = spaces between cells indicating disruption of the monolayer) presented as the mean ± SEM of *n* = 4. Kruskal-Wallis test followed by Dunn’s test with the Bonferroni correction. **(D)** Area of gaps between cells expressed in μm^2^; the area was quantified as the mean of the black area in threshold images from 20 fields per treatment for each n (*n* = 4). ANOVA followed by Tukey’s test. **(E)** FI of CLDN5 per unit area was quantified from 20 cells per treatment for each n (*n* = 4 performed in duplicate) and divided by unit area. f) Morphological characterization of ECs showing the nuclei stained with Hoechst (blue), the F-actin cytoskeleton visualized with Alexa Fluor 488 phalloidin (green), and the tight junction protein CLDN5 visualized with Alexa Fluor 594 (red). Magnification: 60x. Scale bar: 20 μm. The zoomed insets show the condition of the nuclei, the state of the actin cytoskeleton and the distribution of CLDN5. **(G)** Fluorescence profiles (right column) showing the cellular distribution of CLDN5 (red line) in relation to the nucleus (blue line) and cytoskeleton (green line) based on the representative images (left column). The black arrows indicate the tight junction peaks to show the changes in CLDN5 distribution by the treatment. The white line drawn on each cell represents a distance of 50 μm. The data are presented as the means ± SEM, ANOVA followed by Tukey’s test. DMSO = vehicle, Inh = β-secretase inhibitor IV, - or Glu refers to glutamate treatment. All experiments were performed in duplicate. *P < 0.05, **P < 0.01, ***P < 0.001.

To confirm the potential relationship of BACE1 with ECs, ECs were treated with the BACE1 inhibitor at concentrations of 1, 5, 7.5, and 10 μM to measure the resulting cytotoxicity and determine the concentration to be used in subsequent experiments (Supplementary Figure 3). Treatment with 1 μM BACE1 inhibitor exhibited the least toxicity; therefore, this concentration was used for the cell treatments. To characterize the effects of glutamate and the BACE1 inhibitor on the bEnd.3 endothelial cell line, cytotoxicity was measured by determining the percentage of LDH release, and immunolabeling was performed to determine the cell state. In terms of the percentage of LDH released, there were no significant differences among the treatments, but a trend toward increased LDH release in response to glutamate treatment and an apparent reversal of this effect by the inhibitor were observed (Figure 4A). However, the glutamate treatment caused a significant increase in the percentage of condensed nuclei (Figure 4B) and in the number and area of gaps between cells (Figures 4C,D). These effects, except the number of gaps, were significantly reduced by the BACE1 inhibitor. Although the glutamate treatment caused depolymerization of the actin cytoskeleton that was not reversed by BACE1 inhibition (Figure 4F). The tight junction protein CLDN5 was partially recovered by BACE1 inhibition and was distributed to the membrane from the cytoplasm (Figures 4E,G). This finding was confirmed by the CLDN5 FI profile, in which BACE1 inhibition prevented the glutamate-induced increase in CLDN5 immunoreactivity in the cytoplasm, and CLDN5 was partially redistributed to the cell membrane (Figure 4G).

**FIGURE 5:**
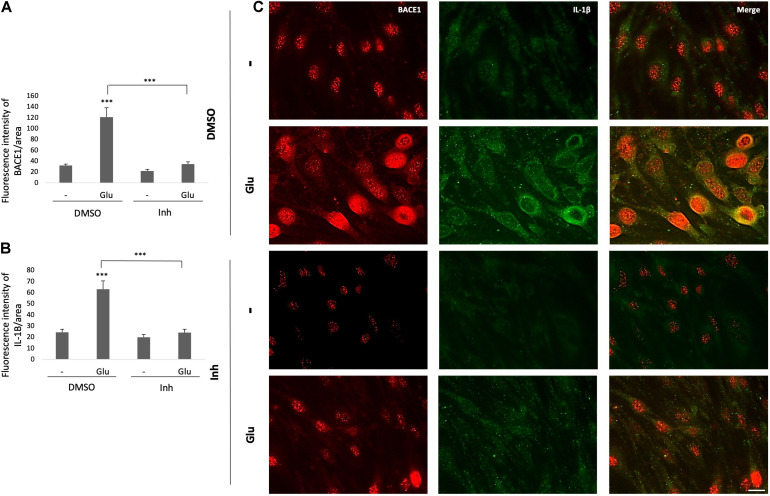
BACE1 inhibitor reduces the glutamate-induced increases in BACE1 IR and the IL-1β inflammation marker in ECs. **(A,B)** Fluorescence intensities of BACE1 and IL-1β, respectively, per unit area, quantified from 20 cells per treatment for each n (*n* = 4 performed in duplicate) and divided by unit area. **(C)** Representative images showing BACE1 (red, visualized with Alexa Fluor 594) and IL-1β (green, visualized with Alexa Fluor 488) expression in ECs (bEnd.3) under different treatments. Magnification: 60x. Scale bar: 20 μm. The data are presented as the means ± SEM, ANOVA followed by Tukey’s test. ***P < 0.001. The bEnd.3 cell cultures were pretreated on DIV 8 with β-secretase inhibitor IV (CAS 797035-11-1, Merck) at 1 μM. Twenty-four hours later, the cells were treated with glutamate at 125 μM for 20 min and were subsequently treated with the inhibitor again. On DIV 10 the cells were fixed for immunofluorescence staining.

Complementarily, BACE1 inhibition decreased the BACE1 IR, which was increased by the glutamate treatment, and reversed the glutamate-induced inflammatory damage as shown by the reduction in the IL-1β IR (Figures 5A,B). BACE1 distribution in the controls appeared to be perinuclear, while IL-1β was localized throughout the entire cell in a largely diffuse pattern with a few brighter spots that looked like vesicles. The glutamate treatment expanded the distribution of BACE1 to the entire cell, although the highest concentration remains in the perinuclear zone where it apparently collocates with IL-1β. BACE1 inhibition upon stimulation with glutamate prevents IL-1β perinuclear concentration, but not in the case of BACE1 which is maintained mainly in such zone but with a diffuse pattern throughout the cells (Figure 5C). Together, these results suggest that BACE1 inhibition maintains the endothelial cell integrity preventing inflammation, cell junction disruption and cell stress by glutamate toxicity.

### BACE1 Inhibition Reverses Astrocytic Reactivity, Causing Cytoskeletal Remodeling and Cell Inflammation

**FIGURE 6:**
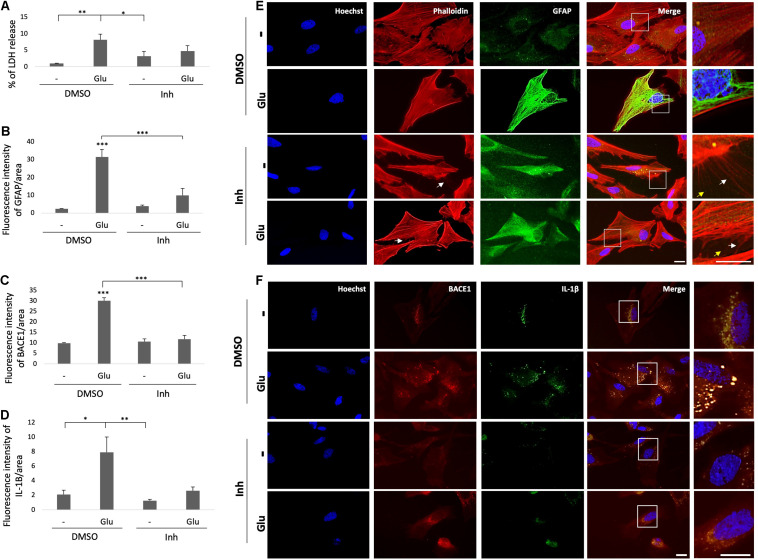
BACE1 inhibition reduces astrocytic reactivity and induces filopodia-like processes. **(A)** Astrocytic cytotoxicity expressed as the percentage of LDH release after 24 h of treatment. The data are presented as the means ± SEM of *n* = 8. Kruskal-Wallis test followed by Dunn’s test with Bonferroni correction. **(B)** FI of GFAP per unit area, quantified from 20 cells per treatment for each n (*n* = 4) and divided by unit area. The data are presented as the means ± SEM. ANOVA followed by Tukey’s test. **(C)** FI of BACE1 per unit area, quantified from 20 cells per treatment for each n (*n* = 4) and divided by unit area. ANOVA followed by Tukey’s test. **(D)** FI of IL-1β per unit area, quantified from 20 cells per treatment for each n (*n* = 4) and divided by unit area. **(E)** Morphological characterization of astrocytes showing the nuclei stained with Hoechst (blue), the F-actin cytoskeleton visualized with Alexa Fluor 594 phalloidin (red), and GFAP visualized with Alexa Fluor 488 (green). Magnification: 60x. Scale bar: 20 μm. In the zoomed insets, the white arrows indicate projections of the actin cytoskeleton, and the yellow arrows indicate extracellular GFAP IR puncta. **(F)** Representative images showing BACE1 (red, visualized with Alexa Fluor 594) and IL-1β (green, visualized with Alexa Fluor 488) expression in primary astrocytes under different treatments. The zoomed insets show the distributions of both proteins and an apparent perinuclear colocalization in vesicles (yellow). Magnification: 60x. Scale bar: 20 μm. DMSO = vehicle, Inh = β-secretase inhibitor IV, - or Glu refers to glutamate treatment. Kruskal-Wallis test followed by Dunn’s test with Bonferroni correction. The data are presented as the means ± SEM, and the experiments were performed in duplicate. *P < 0.05, **P < 0.01, ***P < 0.001.

Reactive astrocytes present morphological and functional changes after injury (Escartin et al., 2021). Therefore, we assessed the morphology astrocytes under stress by glutamate toxicity and BACE1 inhibition. GFAP immunolabeling was performed and the cytotoxicity was measured through determining the percentage of LDH release. The glutamate treatment increased the percentage of LDH released in the treated cells compared with that in the control cells, and BACE1 inhibition did not significantly reduce the percentage of LDH release, although there was a downward trend (Figure 6A). On the other hand, the GFAP FI increased with the glutamate treatment, and BACE1 inhibition reduced the GFAP FI to the baseline level observed in the controls (Figure 6B). The morphological characterization showed the above as well as changes in the microfilaments and intermediate filaments of the cytoskeleton (Figure 6E). BACE1 inhibition induced the production of actin processes which looks like filopodia and depolymerized GFAP, even detected at the extracellular space. These effects were found even when the astrocytes had been treated with glutamate. The IL-1β immunoreactivity showed a clear trend to decrease, although this trend was not significant, whereas the BACE1 protein levels decreased significantly to the baseline levels observed in the controls (Figures 6C,D). The BACE1 immunolabeling was distributed throughout the entire cell, with a stronger bright spot at the perinucleus where it appeared to overlap with IL-1β, whose subcellular localization remains to be confirmed (Figure 6F). In summary, these findings suggest that BACE1 is involved in astrocyte activation and that its inhibition during toxic events could reverse reactive astrocyte and inflammation.

### BACE1-Inhibited Astrocytes Protect Endothelial Cell Integrity by Regulating ZO-1 Distribution and Decreasing Inflammation Caused by Glutamate

**FIGURE 7:**
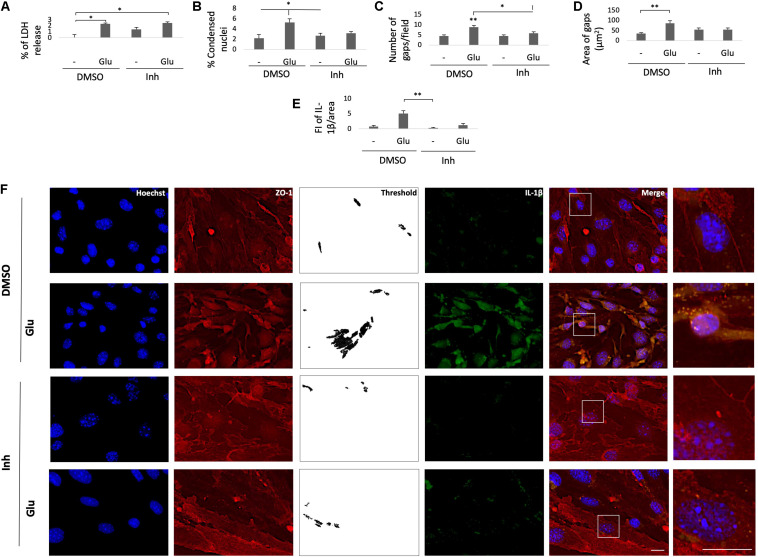
BACE1-inhibited astrocytes protect the integrity of cocultured ECs from damage caused by glutamate. **(A)** Percentage of LDH release by the astrocyte-endothelium coculture after different treatments. **(B)** Percentage of condensed nuclei for each treatment, quantified from 20 fields per treatment for each n. **(C)** The number of gaps per field from threshold images. **(D)** The area of gaps between cells expressed in μm^2^, quantified as the mean of the black area in threshold images from 20 fields per treatment for each n. **(E)** FI of IL-1β per unit area, quantified from 20 cells per treatment for each n and divided by unit area. All data just described are presented as the means ± SEM of *n* = 4. **(F)** Morphological characterization of endothelial cells that were cocultured with primary astrocytes showing nuclei stained with Hoechst (blue), IL-1β visualized with Alexa Fluor 488 (green) and the tight junction protein ZO-1 visualized with Alexa Fluor 594 (red). Magnification: 60x. Scale bar: 20 μm. The zoomed insets show the condition of the nuclei and the distributions of ZO-1 and IL-1β. DMSO = vehicle, Inh = β-secretase inhibitor IV, - or Glu refers to glutamate treatment. All experiments were performed in duplicate. **(A–D)** ANOVA followed by Tukey’s test and **(E)** Kruskal-Wallis test followed by Dunn’s test with Bonferroni correction. *P < 0.05, **P < 0.01.

To validate the effect of BACE1-inhibited astrocytes on endothelial inflammation and apoptosis, astrocyte-endothelial cell coculture was performed under conditions of glutamate toxicity. The levels of ZO-1 and IL-1β and the percentage of LDH release were analyzed to determine the cellular effect. The glutamate treatment significantly increased the percentage of LDH release compared with that of the DMSO control, but this effect was not reversed by coculture with BACE1-inhibited astrocytes. However, the cytotoxicity values that were presented were low since the increase in the cells treated with glutamate was approximately 2.2% compared to that in the control cells (Figure 7A). Accordingly, the glutamate treatment increased the percentage of condensed nuclei by 3% compared to that of the DMSO control, and coculture with BACE1-inhibited astrocytes did not significantly reduce this percentage, although there was a downward trend (Figure 7B). The percentage of condensed nuclei caused by the glutamate treatment in the ECs not cocultured with astrocytes was 38% (Figure 4D), while in the coculture this value was 5% (Figure 7B), indicating that the BACE1-inhibited astrocytes had a protective effect on the endothelium under this stressful condition. In terms of structural damage, both the number and area of gaps between cells were increased by the glutamate treatment, and coculture with BACE1-inhibited astrocytes significantly reduced only the number of gaps (Figure 7C), although there was an evident trend toward decreased gap area (Figure 7D). Regarding inflammation, there was a significant increase in the IL-1β IR of the cells treated with glutamate compared with that of the cells treated with the inhibitor alone, but the IL-1β immunoreactivity of the glutamate-treated cells was not significantly different from that of the DMSO control cells. Coculturing with BACE1-inhibited astrocytes reduced this increase about 4%, which is a clear downward trend (Figure 7E). Furthermore, the basal levels of this cytokine were lower in the cells cocultured with astrocytes than in the cells not cocultured with astrocytes (Figure 5C).

**FIGURE 8:**
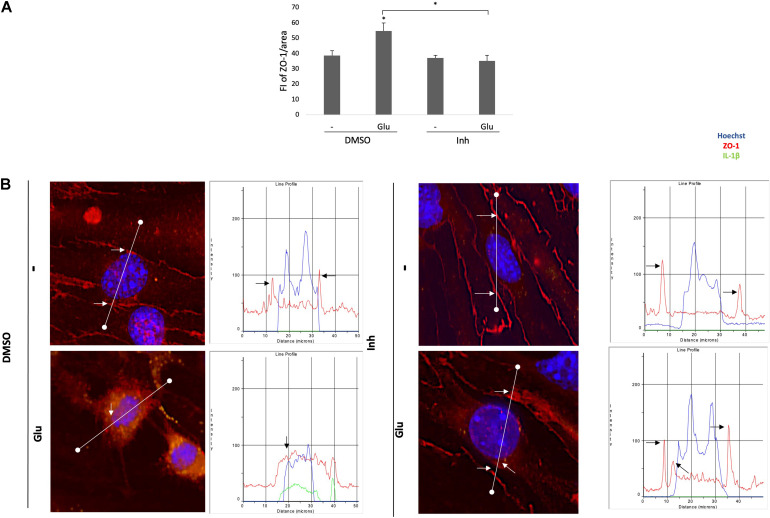
BACE1-inhibited astrocytes reversed the increased IR and cytoplasmic distribution of the ZO-1 protein caused by glutamate in ECs. **(A)** FI of ZO-1 per unit area, quantified from 20 cells per treatment for each n (*n* = 4 performed in duplicate) and divided by unit area. The data are presented as the means ± SEM. ANOVA followed by Tukey’s test. *P < 0.05. **(B)** Fluorescence profiles (right column) showing the cellular distributions of ZO-1 (red line) in relation to the nucleus (blue line) and IL-1β (green line) based on the representative images (left column). The black arrows indicate the tight junction peaks to show the changes in ZO-1 distribution by the treatment. The white line drawn on each cell represents a distance of 50 μm. DMSO = vehicle, Inh = β-secretase inhibitor IV, - or Glu refers to glutamate treatment.

The observed changes in the cytoplasmic distribution and immunoreactivity of CLDN5 induced by the glutamate treatment also occurred with the tight junction protein ZO-1 (Figure 7F). BACE1-inhibited astrocytes reduced the increase in immunoreactivity caused by glutamate at the cytoplasm, and through fluorescence profiles, it was determined that they also cause the redistribution of ZO-1 to the cell membrane, although a portion remained in the cytoplasm, which follows the subcellular distribution described for this protein under nonpathological conditions (Figures 8A,B). Together, these results suggest that BACE1-inhibited astrocytes protect against tight junction damage and inflammation in ECS under glutamate toxicity.

## Discussion

The results of this investigation suggest that BACE1 dysregulation could have a role in the changes observed in the NVU in Alzheimer’s-type dementia. BACE1 augmentation was associated with reactive astrocytes and endothelial disruption in a neurodegenerative environment in the postmortem human brain and *in vitro*. This finding was supported by the increased expression of BACE1 observed in reactive astrocytes associated with PHF-tau and located close to or overlapping with blood vessels in the AD cases respect to the control group. Although, these characteristics were not so evident in the CADASIL cases, there was an increase of BACE1 in subiculum. Complementarily, *in vitro* studies showed that BACE1-inhibited astrocytes reduced the disruption of tight junction (ZO-1^+^) and the increase of IL-1β IF glutamate-induced in cocultured ECs. Therefore, we propose that an overload of BACE1 in reactive astrocytes close to vessels is a triggering factor for neurodegeneration in AD.

It is known that the basal expression of BACE1 is located at CA4 area in human brain and is expressed in dentate gyrus in mice and rat brains (Laird et al., 2005; Xue et al., 2015). This protein is widely described in neurons, but also it has been found in astrocytes (Liang et al., 2020) and brain vessels (Devraj et al., 2016). However, the crucial role of BACE1 in NVU integrity and its implication on dementia has not detailed explored (Huang et al., 2020). In this research, our data support a generalized increase of BACE1 IR in the hippocampus of all dementia cases by immunohistochemistry. Specifically, we confirm that BACE1 was expressed in astrocytes, in concordance with previous studies (Liang et al., 2020). Although we did not observe changes between dementia cases by immunoprecipitation, it was clear by confocal microscopy that BACE1 was overregulated mainly in astrocytes on FAD and SAD groups, with a particular association with vessels in SAD. Additionally accumulated CLDN5 was found according to previous reports (Villar-Vesga et al., 2020; González-Molina et al., 2021). Such BACE1^+^ reactive astrocytes associated to disrupted vessels were also closer but not overlapping with PHF-tau. A potential explanation why vessels are more affected in SAD, it is because SAD is associated to unhealthy lifestyle by metabolic disorders, inducing a slow and chronic impairment of NVU (Cardona-Gómez and Lopera, 2016), being longer this type of vessel affection than on FAD and CADASIL, where the genetic origin starts altering endosomes and endothelial smooth muscle, respectively, with faster and more aggressive progression.

Additional studies have shown BACE1 expression in reactive astrocytes close to β-amyloid peptide (Aβ) plaques in AD brains (Hartlage-Rübsamen et al., 2003). Thus far, it is understood that the astrocytic expression of BACE1 is only relevant to the development of AD if astrocytes also express APP as a substrate of BACE1. For example, primary astrocytes expressing APP generate significant amounts of Aβ peptides (Gray and Patel, 1993; Amara et al., 1999; Beck et al., 1999; Blasko et al., 2000; Docagne et al., 2004). Furthermore, APP is also expressed by reactive astrocytes in experimental models of chronic gliosis (Martins et al., 2001), and the expression of astrocytic APP results in increased generation of Aβ and A4-CT fragments derived from BACE1 activity (Bates et al., 2002; Lesné et al., 2003). However, may exist other explanations to our present results, because BACE1 in addition to neuronal targets; also has targets present in oligodendrocytes affecting myelin production (Pigoni et al., 2016; Fledrich et al., 2018; Zamparo et al., 2019); in astrocytes and ECs (Hemming et al., 2009) have the target LRP1 which is related with lipid and glucose metabolism (Liu et al., 2017; Mao et al., 2017), and the ST6Gal-l protein involved in monocytes trans-endothelial migration through BBB (Deng et al., 2017). Also the target Jagged/Notch in ECs is associated with angiogenesis in AD (Durrant et al., 2020) and astrocytic inflammation (Acaz-Fonseca et al., 2019). Which is in line with our findings about a potential role of BACE1 in astrocyte-endothelium interaction. Complementarily to these data, we have found differential patterns of astrocytes implication in dementia using additional markers such as AQP4, GS1, GLAST1, and for ECs such as Lectin UEA, vimentin, PECAM1 and CLDN5. However, SAD always showed more affection in relationship with disrupted vessels, less thickness, and more production of CLDN5^+^ extracellular vesicles (EVs) [Henao-Restrepo J et al data non published (Villar-Vesga et al., 2020; González-Molina et al., 2021)]. Furthermore, the study from Gonzalez-Molina et al. suggested that EVs from astrocytes of 3xTgAD mice and AD human brain carried out messages that produced endothelial disruption and neuronal retraction (González-Molina et al., 2021).

On the other side, reactive astrocytes express neurofibrillary tangles (NFTs) and have been implicated in neurodegenerative processes (Arima et al., 1998; Botez et al., 1999; Beach et al., 2003; Schultz et al., 2004; Hishikawa et al., 2005; Jellinger and Attems, 2007; Munoz et al., 2007; Lace et al., 2012; Kovacs et al., 2013; López-González et al., 2013; Ferrer et al., 2014). Interestingly, tau hyperphosphorylation and the related cognitive impairment have been reversed by BACE1 targeting in hippocampus, involving Hsc70 and LAMP2 proteins associated to autophagy-related mediated by chaperones (CMA) and increased mTOR activation (Piedrahita et al., 2016), also reducing arachidonic acid, cPLA2 and COX2 proinflammatory signals *in vivo* (Cardona-Gómez and Lopera, 2016). These findings were supported by the reduction of neuronal proinflammatory environment *in vitro* depending on desaturases of fatty acids (Villamil-Ortiz et al., 2016). Furthermore, BACE1 silencing increased HADHA (hydroxyacyl-CoA dehydrogenase/3-ketoacyl-CoA thiolase/enoyl-CoA hydratase) downregulated by glutamate (data non published), suggesting a role of BACE1 in the alteration of fatty acid oxidation. These findings are in concordance with (Sayre et al., 2017), who showed the improvement of fatty oxidation in astrocytes generate neuroprotection. In turn, it make sense if we considered that BACE1 reduction upregulates mTOR signaling and this pathway are involved in lipid metabolism sensing (Menon et al., 2017). The above is possible considering that mTOR does express in astrocytes (Latacz et al., 2015), and mTOR and HADHA potentially interact in proinflammatory environment (Zhang et al., 2017). Therefore, it is important consider a wider role of BACE1 in inflammatory processes even involving lipid metabolism.

Complementarily, *in vitro* results supported our data on human tissue findings. Because glutamate is critical involved in most neurological diseases and must be regulated by recycling in astrocytes (Abbink et al., 2019), where the overload of glutamate produces ATP failure and lipid peroxidation (Sabogal-Guáqueta et al., 2018). Also, a previous study showed that glutamate treatment affected the endothelial resistance in coculture with astrocytes, being more severe without astrocytes coculture, and this physiological measurement was correlated with LDH release and nuclear condensation (Becerra-Calixto et al., 2018). This context could help to explain why the BACE1 inhibitor [Inhibitor IV, specific for BACE1 well characterized previously (Stachel et al., 2004; Ben Halima et al., 2016)], produced astrocytes and ECs protection, since the inhibitor induced changes in the actin filaments with aspect of filopodia-like protrusions, which in astrocytes has been related to release of neurotrophic factors associated to neuroprotection (Posada-Duque et al., 2015). That is very interesting, considering that the overexpression of BACE1 in neurons has been related to decrease F-actin- rich levels through the regulation of B4, an auxiliary subunit of a voltage-gated sodium channel (Miyazaki et al., 2007). Moreover, BACE1 has also been related to the regulation of growth cone collapse (Bãrao et al., 2015). However, additional studies are necessaries to describe the relation between BACE1 and cytoskeletal dynamic.

In addition, BACE1 inhibitor prevented the nuclear condensation and reduce the IL-1β proinflammatory marker not only in astrocytes as well as recovered co-cultured ECs, reducing GAPs that are more related with extravasation and inflammation than with cell death (Hirata et al., 1995; Israelov et al., 2020). In the case of the ECs, our data showed that the percentage of released LDH after glutamate treatment was very low contrasted with nuclear condensation levels, since chromatin condensation occurs in the early stages of excitotoxicity prior to the disruption of the plasma membrane and the release of LDH, that occurs later in an advanced cell detriment (Bezvenyuk et al., 2003; Elmore, 2007). Moreover, the relationship between excitotoxicity and inflammatory phenomena, specifically regarding IL-1β, has been extensively studied (Fogal and Hewett, 2008). Also, tumor necrosis factor-α (TNF-α) has been linked to the disruption of tight and adherent junctions between ECs by increasing BACE1 levels (Deng et al., 2017). And regarding ZO-1, it could be recovered by Sonic hedgehog (Shh) and glial-derived neurotrophic factor (GDNF) released by astrocytes, exerting a protective effect on ECs (Michinaga and Koyama, 2019). However, further experiments are required for a better understanding. Alternatively, the IL-1β increased in glial cells can cause the activation of the inflammatory mediator Cyclooxygenase 2 (COX-2) in neurons, which in turn upregulates BACE1 through Prostaglandin E2 (PGE2) and cAMP in a reciprocal interaction between the two cell types (Wang et al., 2014). Since astrocytes interact not just with neurons but also with ECs, and the molecules mentioned above are conserved in different cell types, it is probable that the increase in astrocyte IL-1β also promotes the activity of cyclooxygenases in ECs, which would result in not only would increase BACE1 but also the vasoconstriction (Wang et al., 2014; Mishra et al., 2016).

Finally, until now, the development of β-secretase inhibitors as therapeutic candidates has been slow because many did not reach the brain, were rapidly transported to the bloodstream, blocked BACE1 in all cells in the brain parenchyma or those are in initial phases (Gosh et al., 2012; Vassar, 2014) and is necessary improve the security and efficacy. There are other alternatives as BBB transporters (Joy Yu et al., 2011), or higher bioavailability of the BACE1 antibody in the parenchyma (Simpson et al., 2014). Other studies have suggested BACE1 in the BBB as a fundamental target for treating the vascular aspects of AD (Roßner et al., 2005). And how had been mentioned before: “Targeting drugs to BACE1-specific extracellular epitopes on the blood-facing luminal side of the endothelium could facilitate drug design, as the need for BBB penetration and resistance to active transport out of the brain can be avoided” (Devraj et al., 2016). In addition, our findings suggest that targeting BACE1 in the NVU in a cell-specific manner (i.e., in astrocytes or vessels) could be converted into new AD therapies that may lead to clinical outcomes. In summary, our work highlights the increased BACE1 expression of reactive astrocytes associated with endothelial disruption as a triggering factor of neurodegeneration in AD, therefore continue being crucial as therapeutical target.

## Author’s Note

BACE1 augment in reactive astrocytes around vessels were associated with Alzheimer’s-type neurodegeneration in the human hippocampus. Increased BACE1 expression was associated with reactive astrocytes and endothelial disruption by glutamate toxicity *in vitro*. In this context, BACE1-inhibited astrocytes protect endothelial cell integrity by preserving ZO-1 distribution and decreasing inflammatory markers.

## Data Availability Statement

The raw data supporting the conclusions of this article will be made available by the authors, without undue reservation.

## Ethics Statement

The studies involving human participants were reviewed and approved by Human Ethical Committee, University of Antioquia. The patients/participants provided their written informed consent to participate in this study. The animal study was reviewed and approved by Ethical Committee of Experimental Animal, University of Antioquia. Written informed consent was obtained from the individual(s) for the publication of any potentially identifiable images or data included in this article.

## Author Contributions

MC-Q, LP-L, GC-G, and RP-D designed experiments, analyzed data, and reviewed and edited the manuscript. MC-Q and GC-G realized human tissue experiments. LP-L performed *in vitro* experiments. CV-L contributed to sampling and diagnosis of human tissue. MC-Q, LP-L, and GC-G wrote the manuscript. All authors contributed to the article and approved the submitted version.

## Conflict of Interest

The authors declare that the research was conducted in the absence of any commercial or financial relationships that could be construed as a potential conflict of interest.

## Supplementary Material

Supplementary Figure 1 BACE1, Phospho-tau, GFAP and CLDN5 immunoreactivities in subiculum area of dementia brains **(A)** Representative image of the subiculum from a human control case. Magnification: 10×. Scale bar: 25 μm. **(B)** Representative images of the BACE1, **(C)** Phospho-tau, **(D)** GFAP, and **(E)** CLDN5 immunoreactivities in subiculum area of human hippocampal tissue. Magnification: 10×. Scale bar: 50 μm. Inset: 25 μm scale bar. The values in the bar graph are expressed as a densitometric percentage of the BACE1 IR in the CA1 area. FAD: familial-type Alzheimer’s disease (presenilin 1 mutation E280A); SAD: sporadic Alzheimer’s disease; CADASIL: autosomal dominant cerebral arteriopathy with subcortical infarcts and leukoencephalopathy. The data are expressed as the means ± SEM. *n* = 4. **p* < 0.05; ***p* < 0.01.

Supplementary Figure 2 BACE1, Phospho-tau, GFAP and CLDN5 immunoreactivities in the CA4 area from demented cases **(A)** Representative image of area CA4 in the hippocampus from a control case. Magnification: 10×. Scale bar: 25 μm. **(B)** Representative images of the BACE1, **(C)** Phospho-tau, **(D)** GFAP, and **(E)** CLDN5 immunoreactivities in the CA4 area of human hippocampal tissue. Magnification: 10×. Scale bar: 50 μm. Inset: 25 μm scale bar. The values in the bar graph are expressed as a densitometric percentage of the BACE1 IR in the CA1 area. FAD: familial-type Alzheimer’s disease (presenilin 1 mutation E280A); SAD: sporadic Alzheimer’s disease; CADASIL: autosomal dominant cerebral arteriopathy with subcortical infarcts and leukoencephalopathy. The data are expressed as the means ± SEM. *n* = 4. **p* < 0.05; *****p* < 0.0001.

Supplementary Figure 3 Cytotoxicity of BACE1 inhibitor at different concentrations. Percentage of LDH released by ECs of the bEnd.3 cell line at BACE1 inhibitor concentrations of 1, 5, 7.5, and 10 μM. The data are presented as the mean of *n* = 3, and the experiment was performed in duplicate. Control = inhibitor vehicle (DMSO).
