# Supplementary figures and images for: Beta-Secretase 1 Underlies Reactive Astrocytes and Endothelial Disruption in Neurodegeneration
Source: Front Cell Neurosci. 2021 May 6;15:656832. doi: 10.3389/fncel.2021.656832 (PMC8136516; doi:10.3389/fncel.2021.656832)

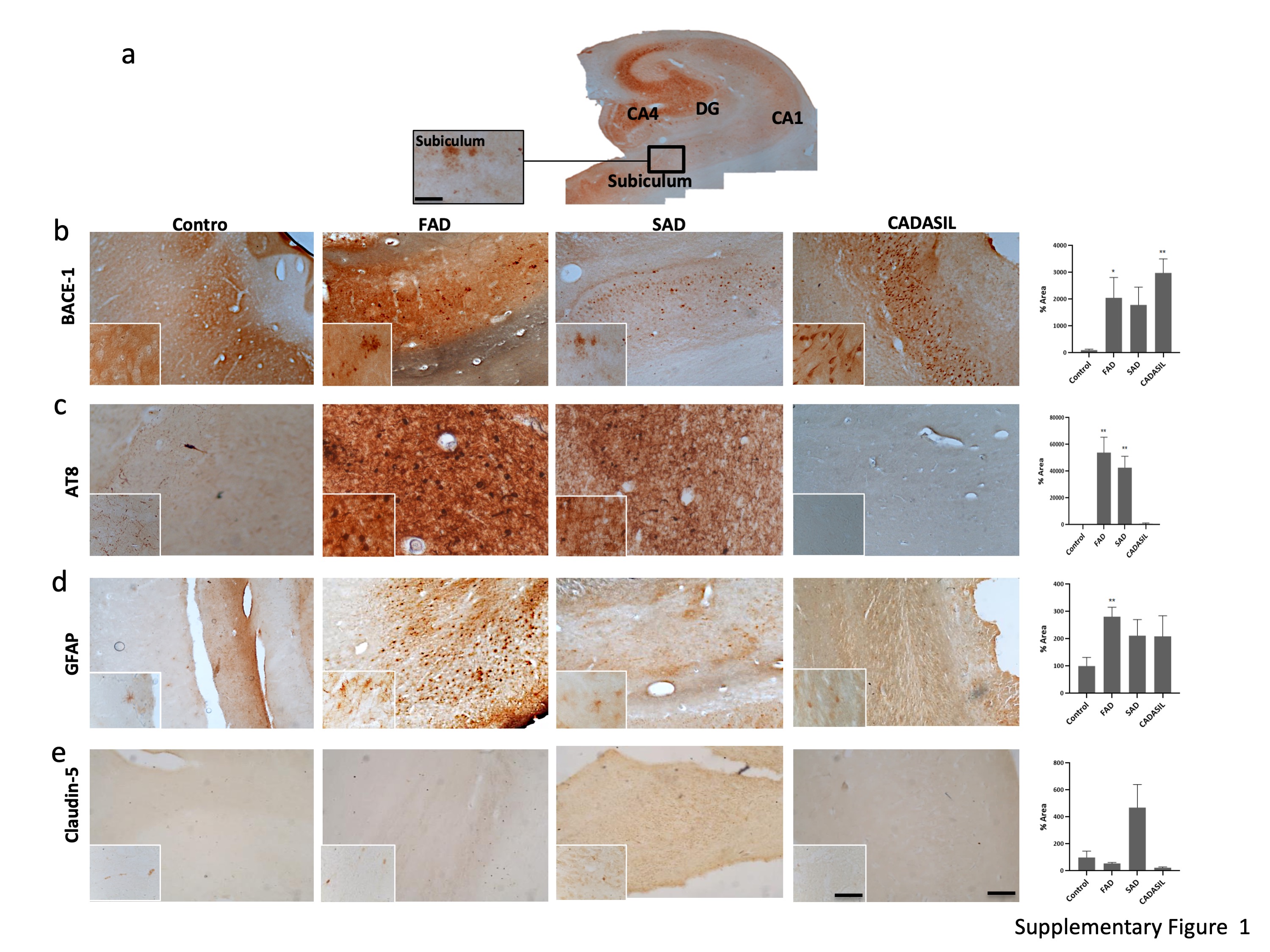

Supplement: Supplementary Figure 1 — BACE1, Phospho-tau, GFAP and CLDN5 immunoreactivities in subiculum area of dementia brains (A) Representative image of the subiculum from a human control case. Magnification: 10×. Scale bar: 25 μm. (B) Representative images of the BACE1, (C) Phospho-tau, (D) GFAP, and (E) CLDN5 immunoreactivities in subiculum area of human hippocampal tissue. Magnification: 10×. Scale bar: 50 μm. Inset: 25 μm scale bar. The values in the bar graph are expressed as a densitometric percentage of the BACE1 IR in the CA1 area. FAD: familial-type Alzheimer’s disease (presenilin 1 mutation E280A); SAD: sporadic Alzheimer’s disease; CADASIL: autosomal dominant cerebral arteriopathy with subcortical infarcts and leukoencephalopathy. The data are expressed as the means ± SEM. n = 4. *p < 0.05; **p < 0.01. [file Image_1.JPEG]

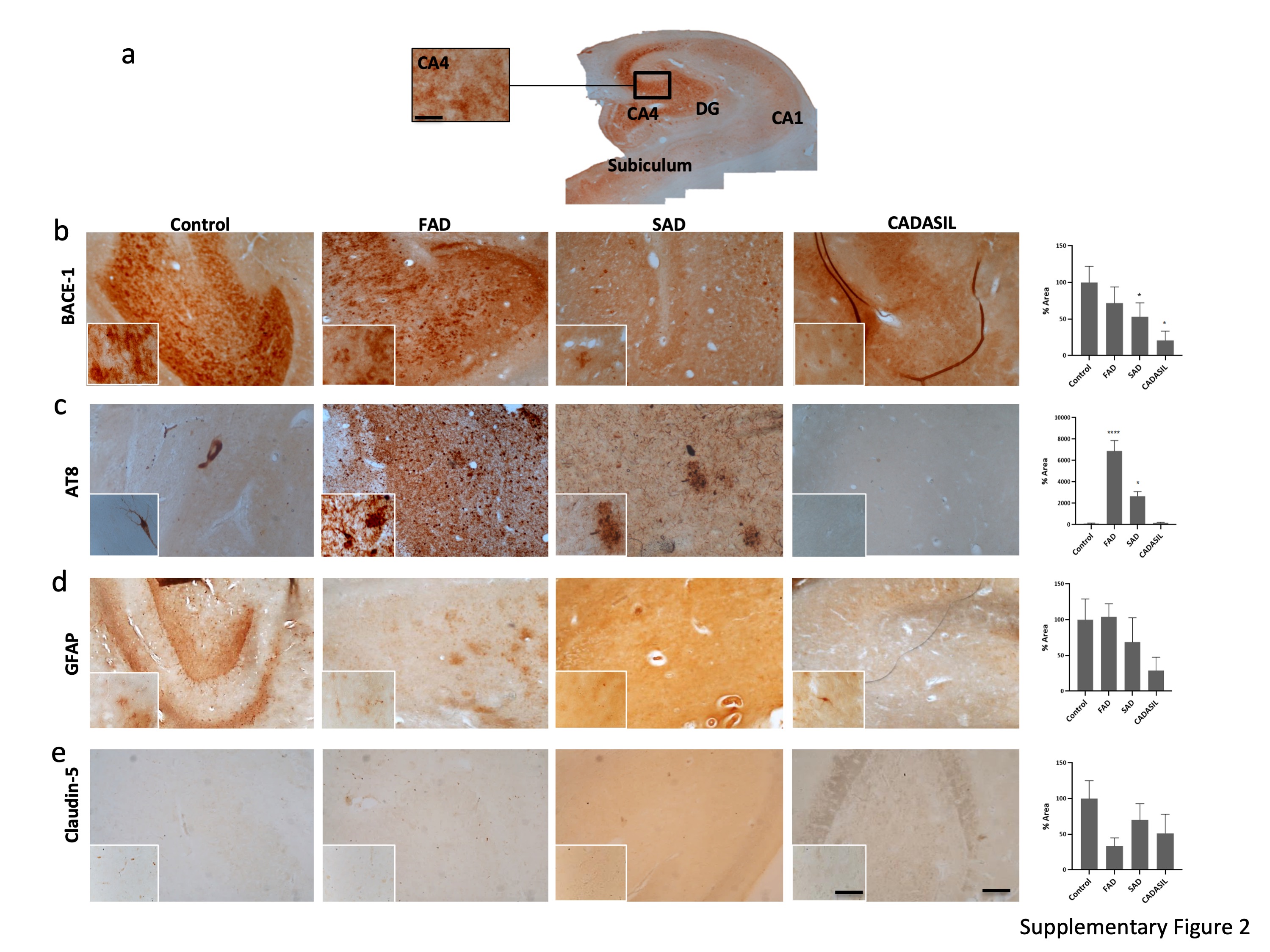

Supplement: Supplementary Figure 2 — BACE1, Phospho-tau, GFAP and CLDN5 immunoreactivities in the CA4 area from demented cases (A) Representative image of area CA4 in the hippocampus from a control case. Magnification: 10×. Scale bar: 25 μm. (B) Representative images of the BACE1, (C) Phospho-tau, (D) GFAP, and (E) CLDN5 immunoreactivities in the CA4 area of human hippocampal tissue. Magnification: 10×. Scale bar: 50 μm. Inset: 25 μm scale bar. The values in the bar graph are expressed as a densitometric percentage of the BACE1 IR in the CA1 area. FAD: familial-type Alzheimer’s disease (presenilin 1 mutation E280A); SAD: sporadic Alzheimer’s disease; CADASIL: autosomal dominant cerebral arteriopathy with subcortical infarcts and leukoencephalopathy. The data are expressed as the means ± SEM. n = 4. *p < 0.05; ****p < 0.0001. [file Image_2.JPEG]

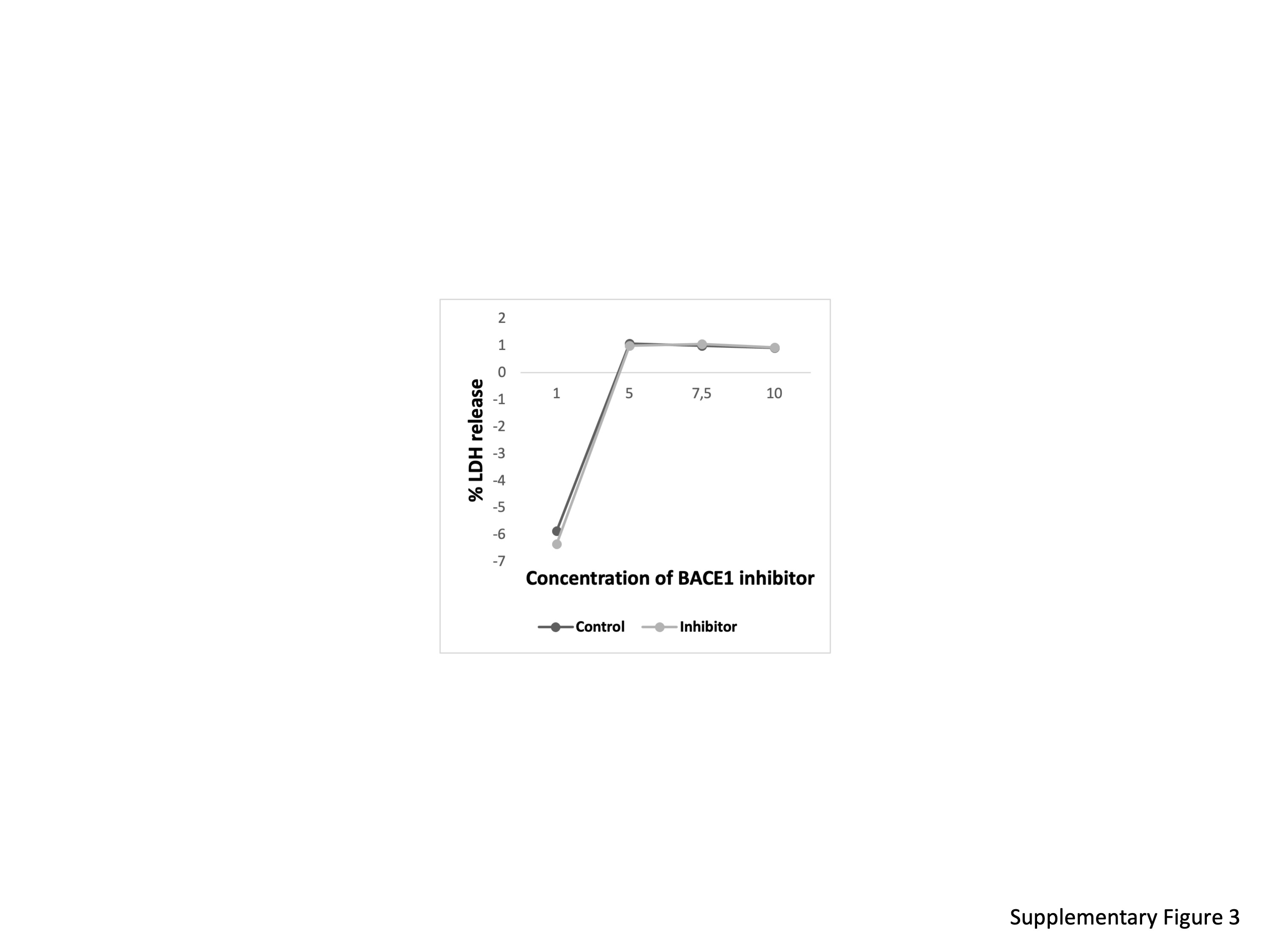

Supplement: Supplementary Figure 3 — Cytotoxicity of BACE1 inhibitor at different concentrations. Percentage of LDH released by ECs of the bEnd.3 cell line at BACE1 inhibitor concentrations of 1, 5, 7.5, and 10 μM. The data are presented as the mean of n = 3, and the experiment was performed in duplicate. Control = inhibitor vehicle (DMSO). [file Image_3.JPEG]
